# Phenotypic Characterization of *Rhodococcus equi* Biofilm Grown *In Vitro* and Inhibiting and Dissolving Activity of Azithromycin/Rifampicin Treatment

**DOI:** 10.3390/pathogens8040284

**Published:** 2019-12-04

**Authors:** Elisa Rampacci, Maria Luisa Marenzoni, Stefano Giovagnoli, Fabrizio Passamonti, Mauro Coletti, Donatella Pietrella

**Affiliations:** 1 Department of Veterinary Medicine, University of Perugia, Via San Costanzo 4, 06126 Perugia, Italy; marialuisa.marenzoni@unipg.it (M.L.M.); fabrizio.passamonti@unipg.it (F.P.); mauro.coletti@unipg.it (M.C.); 2 Department of Pharmaceutical Sciences, University of Perugia, Via del Liceo 1, 06123 Perugia, Italy; stefano.giovagnoli@unipg.it; 3 Department of Pharmaceutical Sciences, Biochemical Sciences and Health Section, University of Perugia, Via del Giochetto, 06122 Perugia, Italy; donatella.pietrella@unipg.it

**Keywords:** biofilm formation, respiratory infection, biofilm dissolution, biofilm inhibition, scanning electron microscopy, fluorescence analysis

## Abstract

Microbial biofilm has been implicated in a wide range of chronic infections. In spite of the fact that *Rhodococcus equi* is a recognized cause of chronic disease in animals and humans, few studies have focused on the sessile phenotype of *R.* *equi*. The aim of this research was to phenotypically characterize the biofilm development of *R. equi* and its answerability for hypo-responsiveness to macrolides and rifampicin. Biofilm formation is initiated by bacterial adhesion to the surface. In this work, the ability of *R. equi* to adhere to the surface of human lung epithelial cells was detected by a fluorometric adhesion test performed on 40 clinical isolates. Subsequently, the capability of *R. equi* to produce biofilm was investigated by colorimetric, fluorescence and scanning electron microscopy analysis, revealing a general slow growth of rhodococcal biofilm and different sessile phenotypes among field isolates, some also including filamented bacteria. Azithromycin treatment produced a higher long-term inhibition and dissolution of *R. equi* biofilms than rifampicin, while the two antibiotics combined boosted the anti-biofilm effect in a statistically significant manner, although this was not equally effective for all *R. equi* isolates. Increasing the MIC concentrations of drugs tenfold alone and in combination did not completely eradicate pre-formed *R. equi* biofilms, while a rifampicin-resistant isolate produced an exceptionally abundant extracellular matrix. These results have strengthened the hypothesis that biofilm production may occur as an antibiotic tolerance system in *R. equi*, potentially determining persistence and, eventually, chronic infection.

## 1. Introduction

Biofilm formation is considered one of the underlying reasons for antibiotic treatment failure [1]. Facultative intracellular bacteria causing chronic infections, such as *Mycobacterium tuberculosis,* are able to embed themselves within an extracellular matrix-enclosed biofilm containing free mycolic acids [2], which is believed to be highly resistant to antibiotics. Recently, *Rhodococcus* species have been added to the list of biofilm forming organisms [3] and are members of the phylogenetic group Mycolata together with the genera *Mycobacterium, Corynebacterium,* and *Nocardia* due to the mycolic acids found in their cell walls. *Rhodococcus equi* is a facultative intracellular pathogen that is well-known as the main etiological agent of subacute/chronic pneumonia in foals and is recognized as a human opportunistic pathogen, responsible for chronic zoonosis, in addition to being a telluric germ that is widespread in the environment. Recently, an increased number of reports have noted the worldwide spread of *R. equi* human infections, especially among immunocompromised subjects [4,5,6,7,8,9,10]. Eighty percent of cases show pulmonary involvement [11], suggesting airborne transmission [12], particularly due to exposure to domesticated horses [13,14]. Indeed, most *R. equi* strains that cause pneumonia in foals and many strains that cause pneumonia in humans carry the plasmidic *vapA^+^* gene [15]. However, since the variant plasmid markers *traA^+^/vapA^−^B^+^, traA^+^/vapAB^−^* have been identified in non-horse hosts intended for human consumption as well as in humans, food-borne transmission seems to be probable [16,17]. Increased concern about *R. equi* infections is ascribed to misdiagnosis as tuberculosis due to the similarities in clinical features, acid-fast staining, and bacterial wall composition with *M. tuberculosis* [7,18]. Notwithstanding increasing evidence of public health risk, little is known about the ability of *R. equi* to establish a sessile microbial community. 

Substantial strain-to-strain variation in growth rates and biofilm formation was observed among high and low passage clinical isolates [19], and one of the few published works concerning *in vitro R. equi* biofilm production reported that 63% of the collected equine clinical samples were populated by biofilm-forming strains at 24 h of incubation [20]. Additionally, *R. equi* isolates causing bacteremia in humans were found to be able to form thick microbial biofilms on the surface of polyurethane catheters [3].

Adhesion of bacteria to host tissue is the first step in the development of biofilm-related infectious diseases [21]. Electron microscopy studies have suggested that facultative intracellular infective microorganisms such as mycobacterial cells may be able to adhere to the lung alveolar epithelium [22] and thus spend part of their pulmonary life-cycle as an extracellular pathogen, potentially forming antibiotic resistant biofilms responsible for disease reactivation. To date, *R. equi* pathogenesis has scarcely been characterized, and appropriate treatments for sessile rhodococcosis have not been established, although the association of a macrolide with rifampicin (RIF) seems effective against planktonic bacteria [23]. The treatment outcome of bacterial biofilms is often unpredictable. Indeed, while appropriate antimicrobial treatments can lead to biofilm eradication [24], sub-effective drug levels can promote biofilm growth [25].

In this work, in order to provide clues supporting the relevance of putative biofilm-related processes in *R. equi* disease, an *in vitro* kinetic analysis of *R. equi* biofilm formation and adhesion to basal human alveolar epithelial cells was carried out. Several equine *R. equi* clinical isolates, including an ATCC cell line, were compared. Moreover, to investigate *R. equi* biofilm formation as a resistance factor, the dissolution and inhibition capacity of the spray-dried azithromycin (AZM) and RIF powder combination, found previously to be effective on a planktonic strain [26], was measured in comparison with using the drugs alone.

## 2. Results

### 2.1. Biofilm Formation and Cellular Adhesion of Fluorescein-Labelled Rhodococcus equi

**Figure 1:**
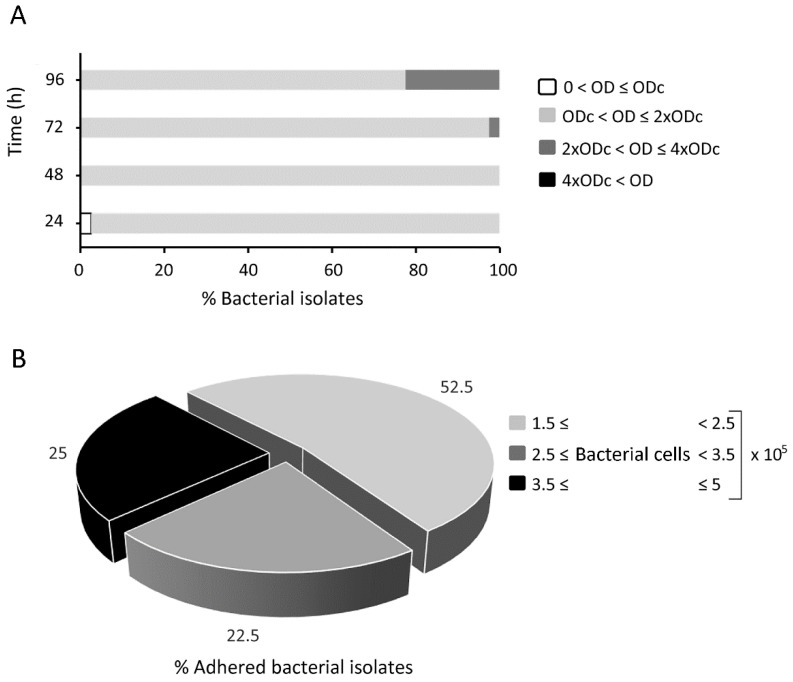
Kinetic growth of rhodococcal biofilms and cellular adhesion of fluorescent rhodococci. (**A**) Growth of rhodococcal biofilm measured at 24 h intervals and categorized as a non-biofilm producer 0 ≤ OD ≤ ODc (negative control absorbance value); weak biofilm producer ODc < OD ≤ 2 × ODc; moderate biofilm producer 2 × ODc < OD ≤ 4 × ODc; and strong biofilm producer 4 × ODc < OD. (**B**) Percentage of adhered bacteria.

After bacteriological and molecular identification and detection of the virulence plasmid *VapA* in 100% (39/39) of the clinical isolates, field bacteria (Re1–Re39) and *R. equi* ATCC 33701 were tested for their capability to form biofilm. Figure 1A summarizes the growth of rhodococcal biofilms, measured at 24 h intervals. No bacterium was described as a strong biofilm producer, i.e., producing more than fourfold higher biofilm mass than the negative control. Nine out of the 40 rhodococci (22.5%), ATCC 33701, Re1, Re4, Re6, Re9, Re19, Re24, Re30, and Re36, formed moderate biofilms at 96 h. Only one bacterium, the isolate Re9, was classified as an early moderate producer at 72 h. At least 48 h of incubation proved to be necessary to confirm all bacteria as biofilm forming organisms through crystal violet biofilm assay.

In an effort to investigate the capacity of bacteria to adhere to the biotic surface, a fluorescence-based adhesion assay was performed on pulmonary alveolar epithelium. Figure 1B shows the percentage of adhered bacteria. All fluorescein isothiocyanate (FITC)-labelled bacteria proved to be able to adhere to the pulmonary basal alveolar epithelium, suggesting the presence of potential cellular receptors involved in *R. equi* adhesion. *R. equi* adhered to A459 monolayers over a percentage range of between 1.5% and 5% of the initial inoculum (equal to 1 × 10^7^ bacterial cells). Accordingly, the number of adherent bacteria, obtained by interpolating the medium intense fluorescence of each isolate with the relative calibration curve, was included in the range of (1.5–5) × 10^5^ bacterial cells. The average percentage adhesion of the positive control *Staphylococcus aureus* ATCC 25923 was equal to 1.3% ± 0.2%. Adhesion percentages of all tested rhodococci and the coefficients of determination (r^2^) associated with the calibration curves plotted for all bacteria in each biological replicate are reported in Supplementary Table S1. The coefficients of determination (r^2^) of the replicates were within the reasonable value range of 0.98–0.9999. A statistically significant correlation was found between the adhesion capability and biofilm produced at 96 h by the Spearman rho test (*p* ≤ 0.039), whereas no statistical significance was observed at 24, 48, and 72 h of biofilm formation (*p* ≥ 0.123).

### 2.2. Minimum Inhibitory Concentration of Antibiotics against Rhodococcus equi Isolates

In this study, the first-line drugs AZM and RIF were tested alone and in the exact drug molar ratio 2:1 for the AZM/RIF combination, which was previously found to be synergistically bactericidal against planktonic *R. equi* and effective against a *R. equi*-infected intracellular model long-term [26], to investigate their minimum inhibitory concentration (MIC) against planktonic *R. equi* and their anti-biofilm properties. As a result of the increased solubility and dispersibility of the excipient-free spray-dried formulation, no organic solvent was used so as to test the drug itself and the combination in an exact drug molar ratio. All bacteria were susceptible to the antimicrobials tested, except for Re4 and Re2—moderate and weak biofilm producers, respectively—which were resistant to RIF. The AZM/RIF 2:1 MIC halved compared to RIF, the most potent compound, against 16/40 rhodococci, including 5/9 moderate biofilm producers: ATCC 33701, Re1, Re9, Re19, and Re24. The MIC values for all the tested treatments are summarized in Table 1.

### 2.3. Long-Term Antimicrobial Prevention of Rhodococcus equi Biofilm

The antibiotic inhibition of the moderate biofilms produced by *R. equi* isolates Re1, Re4, Re9, Re19, Re24, and ATCC 33701 was evaluated after 96 h of incubation by spectrophotometric determination by performing the crystal violet assay. 

Treatments consisted of AZM and RIF alone and AZM/RIF 2:1 at the MIC value. Additionally, to thoroughly investigate the potential boosting effect exerted by the drug combination, AZM and RIF were tested alone at the MIC value acquired in combination (MIC_in combination_).

As detailed in Table 2, AZM demonstrated a dose-dependent efficacy boosted by the combination with RIF. Indeed, AZM at the MIC concentration proved to be the most active long-term antibiotic against *R. equi* biofilm (*p* ≤ 0.029), while AZM alone at the MIC_in combination_ inhibited the biofilm mass to a maximum of 47.1%. AZM/RIF 2:1 proved clearly to be the most effective long-term prevention treatment, second only to AZM, against three out of the six bacteria tested (*p* ≤ 0.007), pointing out that the dosage of AZM in the antibiotic combination is at least fourfold lower than that of the AZM MIC and equal to the AZM MIC_in combination_ (Table 1). Finally, RIF alone at the MIC_in combination_ showed poor inhibition, with a biofilm mass reduction of between 1.1–15.2%, with no statistically significant differences observed from untreated controls. Sub-inhibitory concentrations of AZM and RIF (MIC acquired in combination) did not seem to stimulate biofilm production.

### 2.4. Kinetic Dissolution of Pre-Formed Rhodococcus equi Biofilm

Drugs were tested for their capability to dissolve pre-formed moderate biofilms by the *R. equi* isolates Re1, Re4, Re9, Re19, Re24 and ATCC 33701 over time. Treatments consisted of AZM and RIF alone and AZM/RIF 2:1 at the MIC and 10 × MIC. As above, to thoroughly investigate the potential boosting effect exerted by the drug combination, AZM and RIF were tested alone at the MIC value acquired in combination (MIC_in combination_) and at 10 × MIC.

The results suggest that

(1) No antimicrobial molecule, alone or in combination, is able to completely eradicate a pre-formed biofilm in the present experimental setup;

(2) The dissolution power is lower at 24 h post treatment (no statistically significant difference was found among treatments and the untreated control) than at longer times, particularly after 72 h of antibiotic application, as detailed in Supplementary Table S2;

(3) Basically, AZM alone and in combination with RIF dissolved the *R. equi* biofilm better than RIF monotherapy. Interestingly, AZM/RIF 2:1 at the MIC was as effective against *R. equi* ATCC 33701 biofilm (*p* = 0.011) as AZM alone at 10 × MIC.

**Figure 2:**
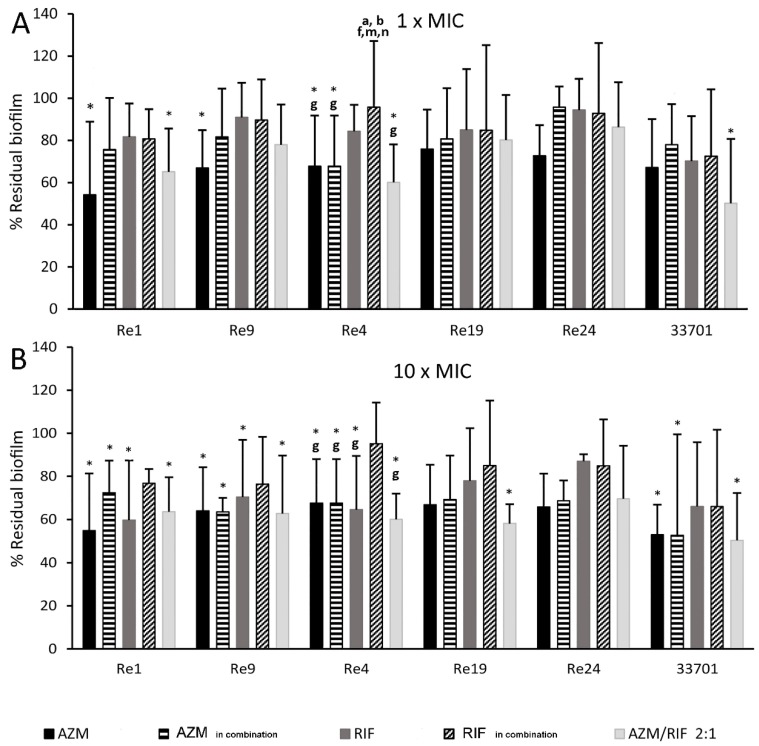
Biofilm dissolution at 72 h post treatment. Results are expressed as the percentage of biofilm residual mass ± SD upon treatment at (**A**) the minimum inhibitory concentration (1 × MIC) and (**B**) 10 × MIC with azithromycin (AZM), rifampicin (RIF), and the AZM/RIF combination at a ratio of 2:1 with respect to untreated controls. AZM and RIF were also tested alone at the MIC value acquired in the combination (MIC_in combination_). Statistical evaluation was performed through ANOVA with post-hoc Tukey HSD and Bonferroni tests. *p* < 0.05 * vs. untreated, **^a^** vs. AZM MIC, **^b^** vs. AZM 10 × MIC, **^c^** vs. AZM MIC_in combination_, **^d^** vs. AZM 10 × MIC_in combination_, **^e^** vs. RIF MIC, **^f^** vs. RIF 10 × MIC, **^g^** vs. RIF MIC_in combination_, **^h^** RIF 10 × MIC_in combination_, **^m^** vs. AZM/RIF 2:1 MIC, **^n^** vs. AZM/RIF 2:1 10 × MIC.

The antimicrobial combination at 10 × MIC had a statistically significant effect, even against Re19 (*p* = 0.022), whereas AZM and RIF alone failed at all concentrations. In turn, no significant biofilm dissolution enhancement was observed when using the drug combination against Re4, a RIF-resistant isolate, Re9, and Re24. Finally, at 72 h post-treatment, RIF alone at 10 × MIC dissolved Re4, Re9, and Re1 biofilm (*p* ≤ 0.035) in a manner comparable to AZM and the antimicrobial combination. The results at 72 h post-treatment are compared in Figure 2.

### 2.5. Biofilm Fluorescence Measurement

**Figure 3:**
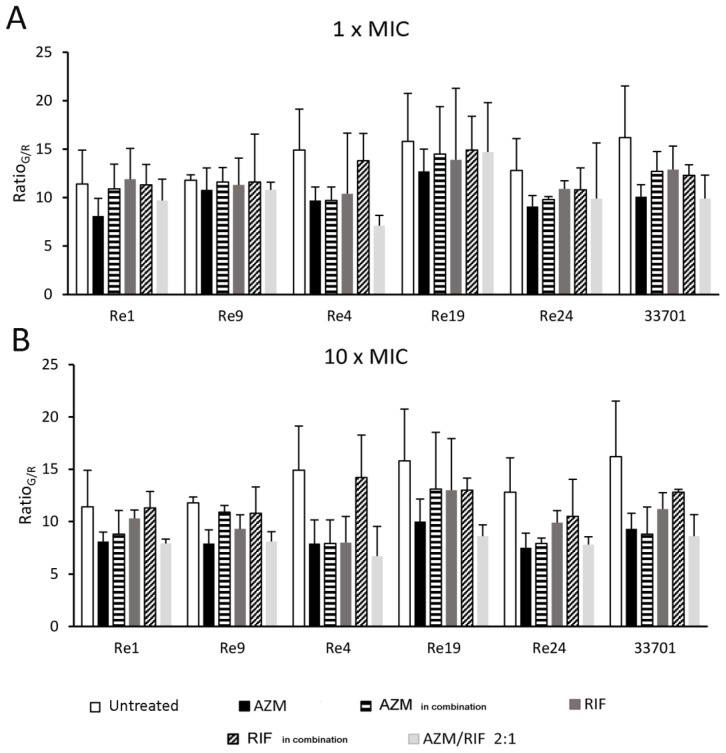
Quotient of the mean fluorescence intensity of green and red light emissions (Ratio_G/R_) that emerged from the LIVE/DEAD biofilm viability assay for the evaluation of the antimicrobial dissolution capability. (**A**) Azithromycin (AZM) and rifampicin (RIF) were tested alone and at a 2:1 combination (AZM/RIF 2:1) at the minimum inhibitory concentration (1 × MIC) and (**B**) 10 × MIC. AZM and RIF were also tested alone at the MIC value acquired in combination (MIC_in combination_). Green fluorescence was detected by setting the excitation/emission wavelengths at 485/530 nm while red fluorescence was set at 485/630 nm.

**Figure 4:**
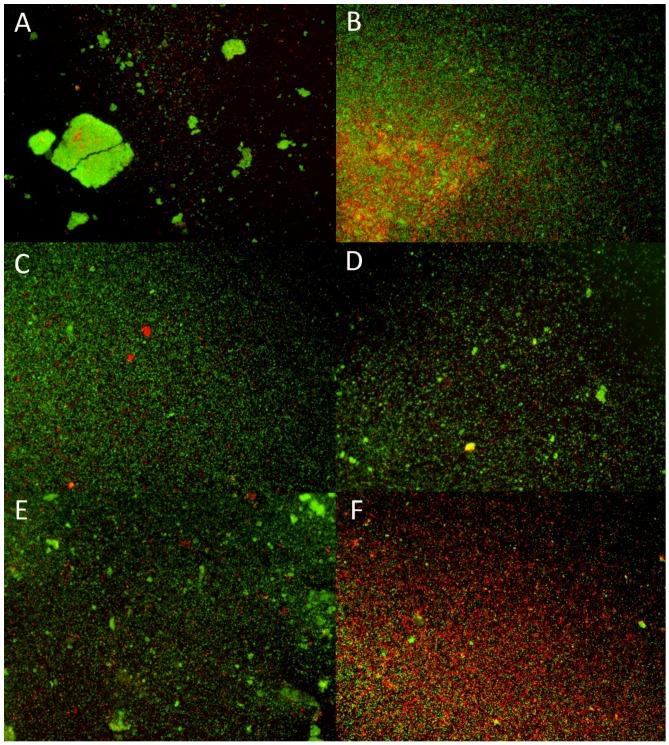
Fluorescent biofilm images of the *Rhodococcus equi* isolate Re19 captured by inverted fluorescence microscope. (**A**) Untreated sessile aggregates; biofilm treated for 72 h with (**B**) azithromycin (AZM) at 10-fold the minimum inhibitory concentration (MIC); (**C**) AZM at 10 × MIC acquired in combination; (**D**) rifampicin (RIF) at 10 × MIC; (**E**) RIF at 10 × MIC acquired in combination**;** (**F**) AZM/RIF 2:1 combination at 10 × MIC.

Ratio_G/R_, a measure of the quantitative relation between green/red fluorescence, depicts the viability of sessile bacterial cells upon antimicrobial dissolution treatments. All ratios are illustrated in Figure 3. Overall, the resulted ratios reflect the measurement of biofilm mass obtained by crystal violet biofilm dissolution assay, confirming AZM and AZM/RIF 2:1, both at a concentration of 10 ×, as the most effective, though not completely eradicating, dissolving treatments. Figure 4 shows the remarkable killing activity of AZM/RIF 2:1 at 10 × MIC against sessile Re19 (F), while other antimicrobial treatments appeared to be ineffective (B-E) compared with untreated control forming biofilm aggregates. This finding portrays the Ratios_G/R_ reported in Figure 3. Additionally, while the Ratios_G/R_ of AZM and AZM/RIF 2:1 at 10 × MIC were very similar, the AZM concentrations were at least fourfold lower in the microparticle combination than for AZM alone.

### 2.6. Structure of Rhodococcus equi Biofilms Captured by Scanning Electron Microscope

**Figure 5:**
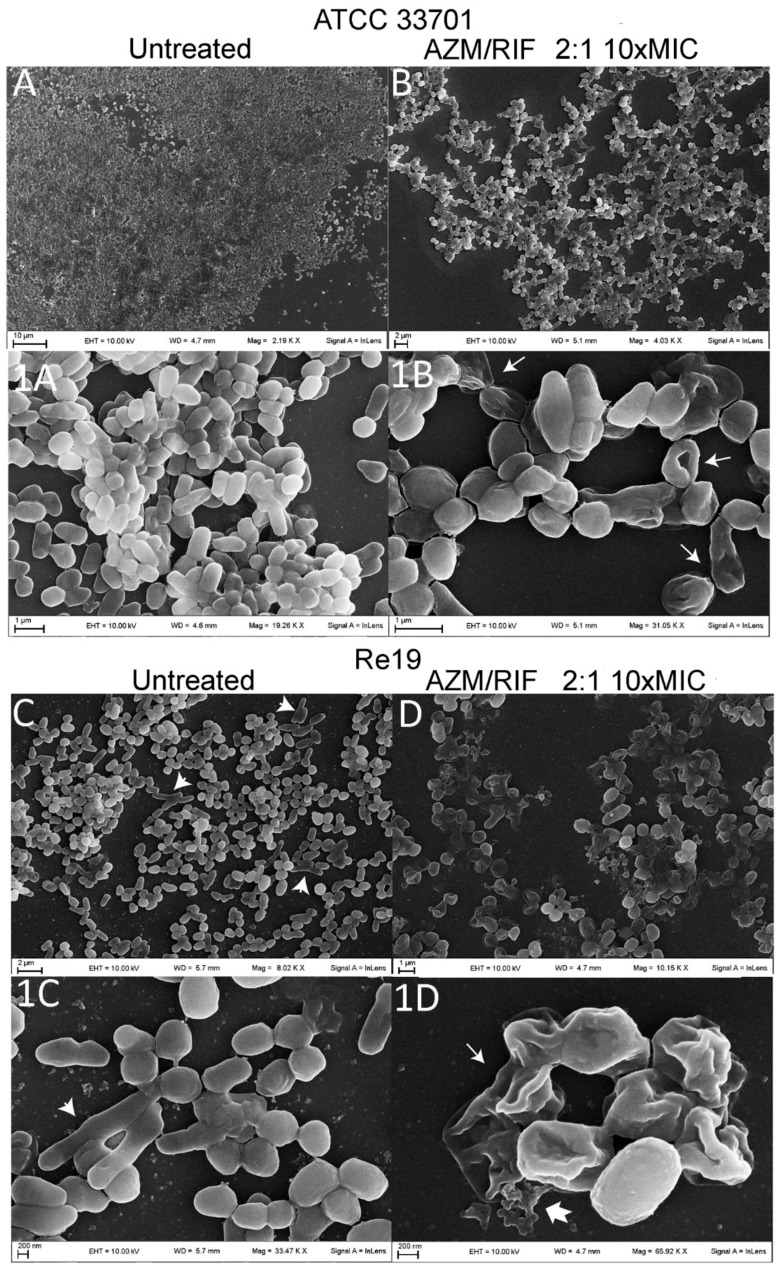
Scanning electron microscopy visual features upon treatment of biofilms of *Rhodococcus equi* ATCC 33701 (**B,1B**) and the clinical isolate Re19 (**D,1D**) with the 2:1 combination of azithromycin and rifampicin at 10-fold the minimum inhibitory concentration compared to untreated biofilms (**A,1A,C,1C**). Measurements were performed at 10 kV and reported at magnifications of 2.2–65.9 kX. Short arrows indicate filamented rhodococci; long arrows indicate damaged bacteria, which appear as empty cells with intact but collapsed structures (ghost cells). The notched arrow refers to the release of intracellular vesicular components from ghost cells.

Further details of the biofilm structural characterization were obtained by scanning electron microscopy (SEM), which highlighted different features depending on the bacterial type observed. When not treated *in vitro* (Figure 5(A,1A)), *R. equi* ATCC 33701 formed a uniform and compact biofilm composed of a thin and minimally fibrous extracellular matrix where the typical coccobacillary shape was clearly preserved. Otherwise, coccobacilli mixed with elongated bacterial cells—evidence of a stressed condition—characterized the untreated biofilm of planktonically drug susceptible *R. equi* clinical isolates, such as Re19, as shown in Figure 5(C,1C).

**Figure 6:**
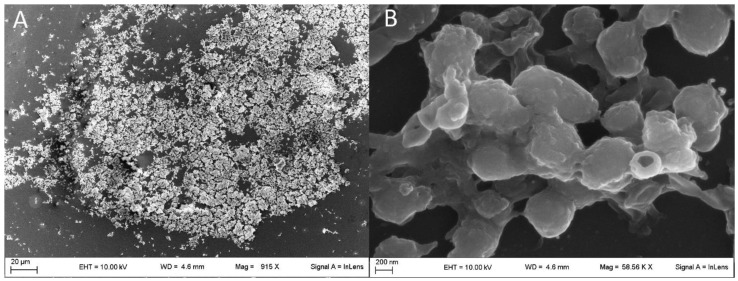
Scanning electron microscopy images showing untreated biofilm formed by the rifampicin-resistant *Rhodococcus equi* isolate Re4. Measurements were performed at 10 kV and reported at (**A**) 915 X and (**B**) 58.6 kX.

As experimentally demonstrated above, treatments with AZM and RIF alone and in combination at ten-fold the MIC value did not totally dissolve the rhodococcal biofilms. However, the antimicrobial challenge damaged the biofilm-enclosed bacterial cells, provoking areas of biofilm detachment and craterization, causing the formation of filamented cells and collapse of bacterial walls. Collapsed cells maintained their structure although many bacteria lost their intracellular components (ghost cells). Such SEM observations are shown in Figure 5(B,1B,D,1D). The RIF-resistant isolate Re4 showed a more copious and fibrous extracellular matrix, connecting bacteria of regular shape in thick sessile aggregates (Figure 6).

## 3. Discussion

### 3.1. In Vitro Model of Rhodococcus equi Biofilm Growth

Biofilm research is crucial to tackle chronic diseases that are notoriously related to bacterial communalism. Bacteria are able to persist in biotic and abiotic environments in sessile aggregates and to resist antimicrobial treatment. To date, poor literature reporting the capacity of *R. equi* to aggregate in a self-produced matrix has been available. *R. equi* seems to be able to produce biofilms, although some clinical isolates have been found to be non-biofilm producers at 24 h of incubation [20]. The kinetic crystal violet assay performed in this work demonstrated that 100% (40/40) of the rhodococci evaluated were biofilm producers with a suggested biofilm production time of at least 48 h, while incubation for longer than 72 h allowed more abundant biofilm development. This fits well with a recent study that did not find significant differences in biofilm formation among *R. equi* isolates at 24 and 48 h [19]. Interestingly, none of the strains were found to be strong biofilm producers. The reason for this may be found in either the manifested slow growth of *R. equi* [27] or sub-optimal *in vitro* growth conditions. A different scenario may occur *in vivo* where biotic and/or abiotic determinants might induce bacterial aggregation during the pathological and environmental life cycle of *R. equi*. It would be interesting to verify the formation of biofilm in an *in vivo* model, but the time span of chronic infection makes it difficult to maintain and control [28]. Therefore, in spite of the fact that compliance with *in vivo* observations is yet to be established, *in vitro* assays remain key methods for preliminarily screening microbial biofilms, allowing the adhesion capability to surrounding surfaces, the production of an extracellular matrix, and the efficacy of antimicrobial molecules to prevent or disperse biofilms to be accessed [29]. 

### 3.2. Putative Pathogenic Significance of Rhodococcus equi Adhesion to Lung Alveolar Epithelium

Surface attachment is the first necessary step for the formation of biofilm on biotic surfaces, after which the growth of the biofilm matrix makes the attachment irreversible, perpetuating the infection [21]. *R. equi* clinical isolates appear to be able to adhere to the pulmonary epithelium in a manner comparable to that of *S. aureus*, which is responsible for serious pneumonia in humans and animals, often by adhering to lung epithelial cells [30] and forming biofilms [31]. After inoculating 1 × 10^7^ bacterial cells, the quantity of rhodococci adhering to the A549 cellular monolayer was >10^5^, a bacterial dose supposedly high enough to cause severe acute pneumonia in neonatal foals, while lower infecting doses have been correlated with the development of insidious disease [32]. Despite the significant association between biofilm production and adhesion profile suggesting a direct correlation, the putative pathogenic significance of this finding remains unknown. Additionally, given that our findings are based on the use of heat-inactivated bacteria, the results from such analyses should be treated with caution, even if the difference in adhesion capability shown by heat-treated and non-heat-treated *R. equi* ATCC 33701 appears to be slight. Heat treatment prevents fluorescence variability due to replicative processes, and it is commonly applied on several bacterial species for surface adhesion characterization [33,34]. However, many questions on the surface binding mechanisms of *R. equi* still need clarification.

### 3.3. Macrolides and Rifampicin versus Biofilm: A Controversial Issue

Azithromycin is intracellularly effective against *R. equi*, infecting macrophage-converted THP-1 cells in a concentration-dependent manner [26]. There is evidence that such an activity affects long-term *R. equi* biofilm formation as well. Indeed, rhodococci treated preventively with anti-rhodococcosis first-line antibiotics produced a biofilm mass inversely proportional to the AZM concentration. Testing drugs at sub-inhibitory concentrations is useful for evaluating a potential stimulatory effect of antibiotics on bacterial biofilm production. Such a phenomenon was noted previously in relation to several antibiotic classes and various bacterial species [35,36]. Particularly, reports concerning the AZM influence on biofilm production are controversial. Sub-inhibitory dosage of this macrolide has the ability to retard *Pseudomonas aeruginosa* biofilm for up to 48 h [37], while the same drug is able to induce biofilm formation at lower concentrations [25]. Exploring such aspects is crucial for establishing an effective therapeutic regimen and thus preventing activation of biofilm production due to premature discontinuation of antimicrobial therapy or excessively low drug dosages at the infection site. Interestingly, we noted that sub-MIC concentrations of AZM—equal to the MIC value acquired in combination (comprised between 1/4–1/16 AZM MIC)—did not stimulate biofilm formation. On the contrary, such dosages showed various degrees of biofilm inhibition depending on the *R. equi* strain tested, different from what was observed on *P. aeruginosa* where 48 h exposure induced a resistant phenotype and strong biofilm formation [37]. Long-term RIF monotherapy at sub-inhibitory concentrations (RIF MIC_in combination_) did not prevent bacterial biofilm formation. Moreover, in contrast with earlier findings against staphylococcal biofilms [38], RIF did not seem to stimulate biofilm production.

The enhancing effect of the AZM/RIF combination in inhibiting and dissolving the rhodococcal biofilm was not equally effective for all *R. equi* isolates. The recorded discrepancies were ascribed to unveiled differences among the bacteria tested. The treatment of bacterial biofilms requires tissue-penetrating antibiotics. Macrolides as well as rifamicins are listed among the molecules that better penetrate in tissues and cells [39]. It is assumed that AZM and RIF, particularly in combination, might have the potential to effectively treat rhodococcal biofilms. However, increasing the concentration of these drugs alone and in combination to ten-fold the MIC did not completely eradicate pre-formed *R. equi* biofilms. This result strengthened our hypothesis that biofilm production may occur as an antibiotic tolerance system in *R. equi* infections. There is limited data on the anti-sessile activity of AZM against Gram-positive bacteria, suggesting a lower anti-biofilm activity than that of clarithromycin [40], the other new generation macrolide. However, a statistically significant reduction of *R. equi* sessile aggregates emerged from our data, particularly when treated with AZM and AZM/RIF 2:1 over a prolonged period of time. These results are in good agreement with a previous study [20] which, however, employed different methodological approaches. On the other hand, the role of RIF against *R. equi* biofilm is controversial. Our data agree fairly well with those of previous studies [41] and further support the use of RIF as an adjunct treatment in place of monotherapy. 

As detailed above, the LIVE/DEAD biofilm viability assay was performed to further compare the anti-sessile activity of AZM and RIF. This experimental method is based on the use of the combination of SYTO9 and propidium iodine. Therefore, it is useful for detecting the live and dead cells in regard to membrane integrity, indirectly undermined by these antimicrobial molecules. Although it has been reported that viable/dead cells might be detected incorrectly by using these combined fluorophores [42], this method enables comparative screening for antimicrobial substances and has been proven to reflect the results obtained by crystal violet biofilm dissolution assay.

### 3.4. Ultramicroscopic Biofilm Phenotype-Based Assumptions

Despite the extracellular matrix enclosing bacterial cells not dissolving completely, the ultramicroscopic details seen in the SEM revealed that highly concentrated antimicrobial treatment appears to increase the population of dead cells or cells suffering from replicative defects, which assume a distinctive collapsed and/or elongated morphological aspect. Particularly, the elongated shape is a result of the anomalous growth of bacteria that continue to elongate but do not divide. Such a phenomenon—known as “filamentation”—is a typical visual feature of stressed bacteria [43] and even provides evidence of interactions between pathogenic bacteria and their hosts [44]. Indeed, filamented bacteria characterize clinical isolates from antibiotically-treated patients [45]. Curiously, untreated biofilm of full drug susceptible isolates showed a mixed population of regular and filamented cells that were missing in *in vitro* untreated samples of Re4, conjecturing a less intense *in vivo* stress response due to the higher drug resistance. Furthermore, this bacterium demonstrated a particularly abundant production of extracellular matrix that might have played a role in the manifested RIF-resistance upon clinical treatment, while Re2—the other RIF-resistant isolate—produced biofilm weakly. Therefore, as yet, the existence and nature of a relationship between RIF resistance and the increased production of the biofilm extracellular matrix have to be ascertained.

## 4. Conclusions

As stated in the introduction, microbial biofilm research is of major value for combating chronic diseases. Very few studies have focused on the clinical and environmental importance of *R. equi* biofilm, in spite of the fact that *R. equi* disease is recognized as one of the leading subacute/chronic infectious diseases in animals and humans and the ability to form a biofilm could be an important virulence determinant for its persistence as well as its survival in the telluric environment. Despite the pathological significance of our findings and the considerably different sessile phenotypes among the field isolates observed deserving more insightful understanding, this study attempted to address some of the significant aspects of the behavior of *R. equi* in sessile form, so as to establish a starting platform for future studies elucidating the clinical relevance of *R. equi* biofilm.

## 5. Materials and Methods

### 5.1. Bacteria Identification

A total of 40 rhodococci were included in this experimental study: the ATCC 33701 and 39 *R. equi* isolated over the course of spontaneous equine pneumonia from 2004 to 2017 in central Italy. All sampling procedures for bacteriological examination complied with national and European regulations and, due to non-experimental-induced sampling, the present study was not subject to approval by the Ethical Committee of our Institution. Bacterial isolates were labelled from Re1 to Re39 and stored at −20 °C. After thawing, each bacterium was sub-cultured and, following the second passage in 5% defibrinated sheep blood agar, DNA of suspected colonies was extracted to confirm the etiological diagnosis and the presence of the virulence plasmid. Briefly, DNA was extracted from pure plated colonies by suspending bacteria in 500 µL of ultrapure water and then incubating them at 100 °C for 10 min. The bacterial suspension was centrifugated at 14,000 rpm for 10 min, and 400 µL of supernatant containing DNA was collected. A modified nested-PCR protocol was performed to detect the ribosomal component 16S gene and virulence plasmid *VapA* according to published methods [46,47]. The *R. equi* 16S gene was amplified in the first PCR reaction by using the primers f-TCGTCCGTGAAAACTTGGGGC and r-CGACCACAAGGGGGGCCGTAT, while in the nested-PCR, f-GAGGAGCGAAAGCGTGGGTA and r-TTAGCCTTGCGGCCGTACTC were used. The two pairs of PCR primers used to amplify VapA plasmid were f-GGTTCTCGTAACGCTACAATC, r-GGTTCGTCTTTCTGAAGGTT and f-TCGGAACTGCCCGAGAACAT, r-GCTCCCAGAACCGACAATGC in the first PCR reaction and nested-PCR, respectively.

### 5.2. Spray-Dried Antibiotic Microparticles

In this study, the first-line drugs AZM and RIF were tested alone and in combination to investigate their MIC against planktonic *R. equi* and anti-biofilm properties. To increase the drug water solubility and powder dispersibility, commercial antibiotic powders were transformed in microparticle formulations by spray-drying. The drugs were tested alone and in the exact drug molar ratio 2:1 for the AZM/RIF combination which, in a previous study [26], demonstrated a synergistic bactericidal effect against planktonic *R. equi* and long-term activity against a *R. equi*-infected intracellular model. The microparticle dry powders were produced using a Mini Spray-Dryer Model B-290 (Büchi, Milan, Italy) starting from excipient-free drug solutions at a final concentration of 2% w/v in acetonitrile and adopting the following conditions: inlet temperature 75 °C, air flow rate 357 L/h, feed rate 2.5 mL/min, and aspirator rate 20 m^3^/h. The quantification of each antibiotic in the AZM/RIF combination was performed by a previously reported HPLC method [26].

### 5.3. Antimicrobial Efficacy against Planktonic Bacteria

The antimicrobial susceptibility assay was performed according to information provided by the Clinical and Laboratory Standards Institute (CLSI) [48]. A broth microdilution procedure was carried out to define the MIC values of AZM, RIF, and AZM/RIF 2:1 against all planktonic *R. equi* isolates over a dose range of 256–0.015 mg/L/component in serial two-fold dilutions in Cation-Adjusted Mueller Hinton Broth (CAMHB). A dilution was prepared in CAMHB starting from a bacterial suspension at the spectrophotometric 0.5 McFarland standard to obtain a final concentration of 5 × 10^4^ CFU/well. Positive and negative control wells were tested in each plate and then incubated at 37 °C. The standard reading was established at 24 h. The experiments were performed in triplicate and in three independent experiments.

### 5.4. Fluorescence-Based Adhesion Assay on Pulmonary Alveolar Epithelium

#### 5.4.1. Bacteria Labelling

*R. equi* isolates and ATCC 33701 were grown in CAMHB at 37 °C under aerobic conditions. The bacterial cells were resuspended in culture medium at 2 × 10^8^ CFU/mL and centrifuged at 3500 rpm for 10 min. The microbial pellets were washed twice with 5 mL of sterile phosphate-buffered saline (PBS) and then centrifuged at 3500 rpm for 10 min to resuspend the bacteria in 1 mL of sterile PBS. The suspended bacteria were heat-treated at 65 °C for 1 h. After inactivation, the cell wall integrity was confirmed by Gram-staining. To label the bacteria, 100 µL of FITC was added at 0.5 mg/mL to 1 mL of bacterial suspension to give a final FITC concentration of 0.05 mg/mL and this was incubated at room temperature in the dark for 30 min. The FITC-labelled bacteria were washed twice with PBS by centrifugation at 6000 rpm for 7 min. Finally, the bacteria were resuspended in 1 mL of sterile PBS and stored at −20 °C until further analysis. To assess the impact of the heat-treatment on bacterial adherence, a preliminary fluorescence-based adhesion test was performed using simultaneously heat-treated and non-heat-treated ATCC 33701. Heat-treated bacteria showed 0.1 percentage point higher adhesion than non-heat-treated ATCC 33701.

#### 5.4.2. *Rhodococcus equi* Adhesion Assay

The A549 cell line, derived from human lung adenocarcinoma of alveolar basal epithelial cells, was obtained from the American Type Culture Collection (A549 ATCC^®^ CCL-185, Manassas, VA, USA). Cells were maintained in RPMI 1640 medium supplemented with 10% fetal calf serum and 1% penicillin/streptomycin. One hundred microliters of a cellular suspension at 2 × 10^5^ cells/mL was seeded into 96-well plates and incubated at 37 °C with 5% CO_2_ in a humidified incubator. After 24 h, the growth medium was removed and the cellular monolayers were washed once with 100 µL of PBS. The adherence capability of each *R. equi* was examined by adding 1 × 10^7^ of the FITC-labelled bacterial suspension on the A549 monolayers to obtain a final bacterial concentration of 1 × 10^8^ bacterial cells/mL in each well (MOI 1:500). At the end of 30 min incubation at 37 °C and 5% CO_2_ in humidified incubator, the supernatant containing non-adhered bacteria was removed and the pulmonary monolayers were washed once with 100 µL of PBS. Finally, 100 µL of PBS was added, and the fluorescence of adherent *R. equi* was read by fluorometer (Infinite M200, Tecan, Salzburg, Austria), setting the excitation at 485 nm and emission at 530 nm. To measure the adherent bacteria on human alveolar epithelial monolayer, calibration curves were built for all rhodococci by plotting the fluorescence emissions of twelve serial two-fold dilutions of the FITC-labelled bacterial suspensions against the corresponding number of bacterial cells, starting from 1 × 10^8^ bacteria/mL seeded on the A549 monolayer. Fluorometric tests were performed on six replicates in three independent experiments (n = 18). *S. aureus* ATCC 25923 was used as a Gram-positive model for pulmonary biofilm production [49], while sterile PBS was used as a negative control. The number of bacterial cells corresponding to the adherent fraction was derived by interpolation with the relative calibration curve. The results were expressed as the percentage of adherent bacteria compared to the initial inoculum, and the coefficient of determination (r^2^) was evaluated as a measure of goodness of fit of the regression model.

### 5.5. Kinetic Growth of Rhodococcus equi Biofilm and Inhibiting/Dissolving Capacity of the Antimicrobial Treatments

Bacteria were grown in CAMHB at 37 °C to reach a spectrophotometric growth absorbance of 0.15 at OD_600_. To assess the growth of rhodococcal biofilms, the microorganisms were diluted 1:50 in CAMHB, and 200 µL of bacterial suspension was seeded in each well of the 96-well plates and then incubated under static conditions at 37 °C for 24, 48, 72, and 96 h. The medium was not supplemented with glucose as it is unlikely to increase the biofilm formation of *R. equi* clinical isolates [20]. At the end of each incubation time, planktonic bacteria were removed and sessile aggregates were washed with 200 µL of PBS. The formed biofilms were stained by adding 100 µL of a 0.4% w/v crystal violet solution. After washing twice with 200 µL of PBS, the bound crystal violet was solubilized with 100 µL of ethanol for 30 min. The mass of sessile bacteria was measured by reading the absorbance at 570 nm. Each measurement was carried out with six replicates for each incubation time. Results were categorized as weak, moderate, and strong biofilm compared to the OD control (ODc) according to published criteria [50]: non-biofilm producer 0 ≤ OD ≤ ODc; weak biofilm producer ODc < OD ≤ 2 × ODc; moderate biofilm producer 2 × ODc < OD ≤ 4 × ODc; strong biofilm producer 4 × ODc < OD. The described colorimetric biofilm assay was performed in three independent experiments (n = 18). Subsequently, AZM, RIF, and their combination at a ratio of 2:1 were assayed to evaluate their ability to inhibit and dissolve *R. equi* biofilm. For biofilm inhibition assay, bacterial suspensions at OD_600_ 0.15 were diluted 1:25 in CAMHB and 100 µL was incubated together with 100 μL of the treatments in 96-well flat plates at 37 °C for 96 h. To verify the long-term biofilm-dissolving effect of the drugs, 96 h-old biofilms were treated with 200 µL of the treatments and incubated at 37 °C for a further 24, 48, and 72 h. At the end of the incubation period, planktonic bacteria were removed and the formed biofilms were stained as described above. The mass of biofilm was measured by comparing the absorbance values of the treated bacteria versus untreated controls and expressed as the percentage residual mass of the untreated control. The biofilm inhibition and dissolution tests were performed, each with four replicates in four independent experiments (n = 16).

### 5.6. Morphological Characterization of Rhodococcus equi Biofilm

#### 5.6.1. LIVE/DEAD Biofilm Viability Assay and Fluorescence Microscopy

Rhodococcal biofilms were stained in triplicate with LIVE/DEAD BacLight Bacterial Viability Kit (ThermoFisher Scientific, Milan, Italy), consisting of the fluorescent nucleic acid stains SYTO9 and propidium iodine when carrying out biofilm dissolution assay. As above, at the end of the 72 h treatment period, planktonic bacteria were removed and the formed biofilms were washed with 200 μL of PBS. Biofilms were stained with 200 μL of LIVE/DEAD solution, prepared following the manufacturer’s instructions for 15 min at room temperature in the dark. After carefully removing floating bacteria, fluorescent biofilms were washed with 200 μL of PBS. Finally, 100 µL of PBS was added and the fluorescence was measured by a fluorometer. Green fluorescence was detected by setting the excitation/emission wavelengths at 485/530 nm, while red fluorescence was set at 485/630 nm. Experiments were assayed in triplicate and repeated twice. Data were analyzed by dividing the mean fluorescence intensity of green light by red light emissions (Ratio_G/R_). Fluorescent image acquisition was performed by inverted fluorescent microscope using a Nikon Eclipse TF2000-S microscope (Nikon, Milan, Italy).

#### 5.6.2. Scanning Electron Microscopy

SEM was applied to image treated and untreated biofilms of *R. equi* ATCC 33701 and field isolates by using a FEG LEO 1525 high resolution microscope equipped with a GEMINI column (ZEISS, Jena, Germany). Biofilms were grown on sterile round glass slides at the bottom of 24-well plates for 96 h. After removing planktonic bacteria, biofilms were treated with AZM and RIF alone and in a 2:1 combination at 1 and 10 × MIC for 72 h, as described above. At the end of the incubation period, biofilms were washed with PBS and fixed with 2.5% w/v glutaraldehyde for 1 h and dehydrated with ethyl alcohol at 50% for 10 min, 85% for 10 min, 95% for 15 min, and 100% absolute twice for 10 min each. After drying, the biofilms on the round glass slides were placed onto an aluminum stub covered with carbon tape and coated with chromium at 20 mA for 18 s prior to imaging. Measurements were performed at 5 kV and images are reported at magnifications between 915 X and 65.9 kX.

### 5.7. Statistical Analysis

Since histogram evaluation showed asymmetric distribution of bacterial adhesion data, the non-parametric Spearman rho test was applied to measure the correlation between the adhesion profile and biofilm produced at 24, 48, 72, and 96 h. The ANOVA test with post-hoc Tukey HSD and Bonferroni correction was applied to compare treatments in the colorimetric biofilm inhibition and dissolution assays as biological replicates, assessing the biofilm residual masses upon treatment. All analyses were performed at 95% significance level.

## Figures and Tables

**Table 1:** Minimum inhibitory concentrations of azithromycin (AZM), rifampicin (RIF), and the AZM/RIF combination at a 2:1 ratio against *Rhodococcus equi*.

Bacteria	AZM	RIF	AZM/RIF 2:1	Bacteria	AZM	RIF	AZM/RIF 2:1
**Re1**	2	0.125	0.125/0.06	**Re21**	2	0.06	0.125/0.06
**Re2**	1	64	1/0.5	**Re22**	1	0.125	0.25/0.125
**Re3**	2	0.06	0.06/0.03	**Re23**	2	0.25	0.25/0.125
**Re4**	1	128	1.0/0.5	**Re24**	1	0.125	0.125/0.06
**Re5**	1	0.25	0.25/0.125	**Re25**	1	0.125	0.25/0.125
**Re6**	1	0.125	0.25/0.125	**Re26**	1	0.125	0.125/0.06
**Re7**	1	0.03	0.06/0.03	**Re27**	1	0.25	0.25/0.125
**Re8**	1	0.06	0.06/0.03	**Re28**	2	0.25	0.5/0.25
**Re9**	1	0.25	0.25/0.125	**Re29**	2	0.06	0.125/0.06
**Re10**	2	0.06	0.125/0.06	**Re30**	1	0.06	0.125/0.06
**Re11**	1	0.25	0.25/0.125	**Re31**	1	0.06	0.125/0.06
**Re12**	2	0.125	0.25/0.125	**Re32**	2	0.125	0.25/0.125
**Re13**	2	0.125	0.25/0.125	**Re33**	1	0.06	0.125/0.06
**Re14**	2	0.25	0.25/0.125	**Re34**	1	0.125	0.25/0.125
**Re15**	2	0.125	0.25/0.125	**Re35**	1	0.06	0.125/0.06
**Re16**	1	0.125	0.25/0.125	**Re36**	1	0.03	0.06/0.03
**Re17**	1	0.25	0.25/0.125	**Re37**	1	0.03	0.06/0.03
**Re18**	2	0.015	0.03/0.015	**Re38**	1	0.125	0.25/0.125
**Re19**	1	0.125	0.125/0.06	**Re39**	2	0.25	0.25/0.125
**Re20**	1	0.125	0.125/0.06	**33701**	0.5	0.125	0.125/0.06

Results are expressed in mg/L and represent the mean of three independent experiments performed in triplicate.

**Table 2:** Long-term antimicrobial inhibition of *Rhodococcus equi* biofilm formation.

	Re1	Re9	Re4	Re19	Re24	33701
AZM MIC	38.2 *^,b,c,d^	53.6 *^,b,c,d^	48.5 *^,d^	49 *	69.1	44.6 *^,b,c,d^
AZM MIC_in combination_	72.7 ^a^	78.6 ^a^	48.5 *^,d^	80.7	81.1	98.9 ^a^
RIF MIC	78.6 ^a^	78.3 ^a^	49.9 *^,d^	84.5	91.7	97.8 ^a^
RIF MIC_in combination_	84.8 ^a,e^	94.1 ^a,e^	88.1 ^a,b,c,e^	95.6	94.1	98.9 ^a^
AZM/RIF 2:1 MIC	53.2 *^,d^	69.4 *^,d^	48.4 *^,d^	72	84.4	59.4 *

Results are expressed as the percentage (%) residual mass upon treatment with the minimum inhibitory concentrations (MICs) of azithromycin (AZM), rifampicin (RIF), and the AZM/RIF combination at a ratio of 2:1 with respect to untreated controls. AZM and RIF were also tested alone at the MIC value acquired in combination (MIC_in combination_). Post-hoc ANOVA was applied for comparing the treatment groups at the 95% significance level. *p* < 0.05 * vs. untreated, **^a^** vs. AZM MIC, **^b^** vs. AZM MIC_in combination_, **^c^** vs. RIF MIC, **^d^** vs. RIF MIC_in combination_, **^e^** vs. AZM/RIF 2:1 MIC.

## Supplementary Materials

The following are available online at https://www.mdpi.com/2076-0817/8/4/284/s1, Table S1: Bacterial adhesion to A549 lung alveolar monolayers and coefficients of determination r^2^ of the calibration curves obtained for all rhodococci tested., Table S2: Percentage biofilm residual mass after 24, 48 and 72 h dissolving treatment with the minimum inhibitory concentration (MIC) and 10xMIC of azithromycin (AZM), rifampicin (RIF) and AZM/RIF combination at a ratio of 2:1.
