# Supplementary material for: Phenotypic Characterization of Rhodococcus equi Biofilm Grown In Vitro and Inhibiting and Dissolving Activity of Azithromycin/Rifampicin Treatment
Source: Pathogens. 2019 Dec 4;8(4):284. doi: 10.3390/pathogens8040284 (PMC6963269; doi:10.3390/pathogens8040284)
Supplement: Supplementary file 1 [file pathogens-08-00284-s001.pdf]

Supplementary material

Table S1. Bacterial adhesion to A549 lung alveolar monolayers and coefficients of determination r^2 of the calibration curves obtained for all rhodococci tested. The adhesion percentage was calculated as ratio between fluorescence of adherent bacteria and fluorescence of bacteria inoculated ($1x10^7$ bacterial cells).

Bacteria	Adherence (mean % ± S.D.)	r^2	Bacteria	Adherence (mean % ± S.D.)	r^2
**Re1**	2.5 ± 1.2	0.9955 – 0.9998	**Re21**	2.5 ± 0.7	0.9992 – 0.9993
**Re2**	2.2 ± 0.9	0.9825 – 0.9994	**Re22**	2.1 ± 0.4	0.9992 – 0.9994
**Re3**	2.0 ± 0.9	0.997 – 0.9999	**Re23**	1.5 ± 0.3	0.9962 – 0.9992
**Re4**	2.3 ± 1.4	0.9979 – 0.9998	**Re24**	1.9 ± 0.6	0.9963 – 0.9998
**Re5**	1.8 ± 0.5	0.9985 – 0.9997	**Re25**	2.3 ± 0.7	0.9953 – 0.9981
**Re6**	2.3 ± 0.9	0.9947 – 0.9997	**Re26**	2.0 ± 1.0	0.997 – 0.9989
**Re7**	2.7 ± 1.6	0.9979 – 0.9999	**Re27**	2.1 ± 0.5	0.9911 – 0.9998
**Re8**	1.8 ± 0.8	0.9988 – 0.9997	**Re28**	2.7 ± 2.5	0.9948 – 0.999
**Re9**	2.7 ± 1.4	0.9995	**Re29**	2.6 ± 2.0	0.9798 – 0.9993
**Re10**	3.3 ± 1.7	0.9961 – 0.9994	**Re30**	5.0 ± 1.5	0.998 – 0.9998
**Re11**	1.8 ± 0.5	0.9978 – 0.9993	**Re31**	4.9 ± 1.5	0.9988 – 0.9997
**Re12**	1.8 ± 0.2	0.9992 – 0.9997	**Re32**	5.0 ± 1.3	0.9997 – 0.9999
**Re13**	1.5 ± 0.5	0.9917 – 0.9996	**Re33**	4.7 ± 1.0	0.9993 – 0.9994
**Re14**	2.7 ± 1.4	0.98 – 0.9997	**Re34**	4.7 ± 1.5	0.9996 – 0.9998
**Re15**	2.0 ± 0.3	0.9941 – 0.9995	**Re35**	4.7 ± 1.4	0.9986 – 0.9998
**Re16**	2.3 ± 0.5	0.9977 – 0.9999	**Re36**	4.5 ± 1.8	0.9984 – 0.9996
**Re17**	2.4 ± 0.9	0.9984 – 0.9993	**Re37**	4.3 ± 2.2	0.9995 – 0.9996
**Re18**	2.2 ± 1.0	0.9987 – 0.9997	**Re38**	4.4 ± 1.3	0.9996 – 0.9998
**Re19**	1.7 ± 0.3	0.9937 – 0.9989	**Re39**	4.8 ± 0.9	0.9986 – 0.9994
**Re20**	1.7 ± 0.8	0.9992 – 0.9996	**33701**	2.6 ± 1.0	0.9993 – 0.9997

Table S2. Percentage biofilm residual mass after 24, 48 and 72 h dissolving treatment with the minimum inhibitory concentration (MIC) and 10xMIC of azithromycin (AZM), rifampicin (RIF) and AZM/RIF combination at a ratio of 2:1. AZM and RIF were tested alone also at the MIC value acquired in combination ($MIC_{in\ combination}$). Post-hoc ANOVA was applied for comparing the treatment groups at 95% significance level. $P < 0.05$ * vs Untreated, [a] vs AZM MIC, [b] vs AZM 10xMIC, [c] vs AZM $MIC_{in\ combination}$, [d] vs AZM $10xMIC_{in\ combination}$, [e] vs RIF MIC, [f] vs RIF 10xMIC, [g] vs RIF $MIC_{in\ combination}$, [h] vs RIF $10xMIC_{in\ combination}$, [m] vs AZM/RIF 2:1 MIC, [n] vs AZM/RIF 2:1 10xMIC.

	Time (h)	Re1	Re9	Re4	Re19	Re24	33701
**AZM MIC**	24	86.7	95.5	87.2	94.6	83.1	99.1
	48	51.8*	72.2	84.7	78.6	86.7	80.9
	72	54.2*	66.9*	67.7*[g]	75.8	72.7	67.1
**AZM 10xMIC**	24	72.7	93.7	86.4	86.4	82.4	91.9
	48	49.5*,[g]	69.5	75.6	75.3	75.3	68.2
	72	54.8*	64*	67.6*,[g]	66.8	65.8	52.9*
**AZM $MIC_{in\ combination}$**	24	85.4	100	-	95.8	86.7	98.8
	48	72.9	88.5	-	97.5	85.9	87.9
	72	75.6	81.6	-	80.7	95.7	78
**AZM $10xMIC_{in\ combination}$**	24	85.8	95.1	-	93.1	83	92.3
	48	55.9*	66.7	-	77.5	83.4	72.7
	72	72.4*	63.5*	-	69.2	68.7	52.6*
**RIF MIC**	24	93.5	95.8	92.5	98.8	93.4	99.4
	48	71.4	90.9	82.7	84.2	92.2	91.4
	72	81.6	90.9	84.3	84.9	94.4	70.2
**RIF 10xMIC**	24	78.5	93.4	88.1	85	94	97.8
	48	62.8*	82.2	66.1	75.9	84.4	75.3
	72	59.7*	70.4*	64.5*,[g]	77.9	87	66
**RIF $MIC_{in\ combination}$**	24	95.5	98.1	95.5	95.2	93.6	101.5
	48	83.1	91.7	98.2	84.4	95.6	93.1
	72	80.7	89.6	95.7[a,b,f, m,n]	84.8	92.8	72.5
**RIF $10xMIC_{in\ combination}$**	24	83.2	94.9	97.7	87.9	93.7	96.8
	48	70.5	85.2	97.9	82.5	84.7	74.7
	72	76.8	76.4	95.1	85	84.9	66
**AZM/RIF 2:1 MIC**	24	87.4	100	90.6	88.3	89	101
	48	70.8	73,5	75.3	76.2	86.7	75.7
	72	65.1*	77.9	60.1*,[g]	80.2	86.3	50.2*
**AZM/RIF 2:1 10xMIC**	24	78	86.5	87.5	89	80	89.8
	48	54.5*	66.7	76.7	70	71.1	75
	72	63.6*	62.7*	60.1*,[g]	58.1*	69.6	50.3*
